# Genotype based Risk Predictors for Polycystic Ovary Syndrome in Western Saudi Arabia

**DOI:** 10.6026/97320630015812

**Published:** 2019-12-10

**Authors:** Sherin Bakhashab, Nada Ahmed

**Affiliations:** 1 Biochemistry Department, King Abdulaziz University, Jeddah, P.O. Box 80218, Saudi Arabia; 2 Centre of Innovation in Personalized Medicine, King Abdulaziz University, Jeddah, P.O. Box 80216, Saudi Arabia

**Keywords:** Polycystic ovary syndrome, THADA, TOX3, FSHR, YAP1, RAB5B, *HMGA2*

## Abstract

Polycystic ovary syndrome (PCOS) is the most common endocrine disease among premenopausal women. The genetic risk of PCOS in the Saudi population is still unclear. Therefore, it is of interest to study the genotype and allele frequency for six gene variants (THADA rs13429458, TOX3 rs4784165, FSHR rs2268361, YAP1 rs1894116, RAB5B rs705702, and HMGA2 rs2272046) in patients with PCOS in western Saudi population. The study included 95 PCOS patients and 94 normal ovulatory females as controls. Genotyping was performed using TaqMan^™^ real-time polymerase chain reaction assays. There was significant link between the THADA rs13429458 variant and PCOS. Homozygosity in allele A of the rs13429458 variant was correlated with hyperandrogenism (HA) risk. Homozygosity in the T allele of the FSHR rs2268361 variant was associated with normal levels of AMH among non-PCOS women. The THADA rs13429458 and TOX3 rs4784165 variants were significantly associated with the combined oligo/amenorrhea (OA) and polycystic ovarian morphology subgroups while the HMGA2 rs2272046 variant was significantly associated with the combined HA and OA subgroup. Thus, results show the genetic risk of the THADA rs13429458, TOX3 rs4784165, and HMGA2 rs2272046 variants on PCOS patients in the western Saudi population.

## Background

The diagnostic criteria for polycystic ovary syndrome (PCOS), a complex endocrine disorder affecting reproductive-aged women, have evolved since the disorder was first recognized. The National Institutes of Health first defined PCOS as the presence of clinical or biochemical hyperandrogenism (HA) comorbid with oligo/amenorrhea (OA) [1]. The Rotterdam consensus added the polycystic ovarian morphology (PCOM) phenotype, and the diagnosis was redefined as the presence of two out of the three conditions[2]. The Androgen Excess Society subsequently considered HA a key component in PCOS diagnosis [3]. The Rotterdam criteria was endorsed by an Endocrine Society clinical practice guideline [4]. According to the diagnostic criteria, the prevalence of PCOS varies worldwide but is generally 6-20% [5-7]. Although there are no prevalence studies that include the entire kingdom of Saudi Arabia, a study conducted in the city of Madinah found a prevalence of 32.5% [8]. Associated with significant multiple clinical manifestations including reproductive, metabolic, and psychological disorders [9-15], PCOS represents 80% of anovulatory infertility cases [10], and 80-85% of women with clinical HA have PCOS [16,17]. Manifestation of the disorder varies depending upon the particular diagnostic criteria. Patients diagnosed according to the Rotterdam and NIH criteria are at higher risk of developing reproductive and metabolic disorders such as infertility and type-2 diabetes [18-21]. The etiology of PCOS is not entirely clear; however, the disease is primarily attributed to multiple genetic and environmental factors aggravated by obesity [22].Heritability of PCOS has been confirmed through twin, family, candidate gene, and genome-wide association studies (GWAS) [23-27]. Two GWAS conducted within the Han Chinese population identified 15 risk single nucleotide polymorphisms (SNPs) at 11 loci [23,26]. Another large-scale GWAS of European Caucasian woman identified six relevant genetic loci [28]. Recently, four studies of European populations confirmed the association of many loci with PCOS [29-32]. The common SNPs correlating to PCOS in women of Chinese and European ancestry were rs13429458 associated with thyroid adenoma (THADA), rs4784165 in the TOX high mobility group box family member 3(TOX3), rs2268361 in the follicle-stimulating hormone receptor (FSHR), rs1894116 in yes-associated protein 1 (YAP1), rs705702 in ras-related protein 5B (RAB5B), and rs2272046 in high mobility group AT-hook 2 (HMGA2)[23,26,28-32].

The THADA gene encodes the thyroid adenoma-associated protein, expressed in the pancreas, adrenal medulla, thyroid, adrenal cortex, testis, thymus, small intestine, and stomach[33]. It was first identified as a target of 2p21 chromosomal aberrations in benign thyroid adenomas, where it disrupted and fused to an intron of peroxisome proliferator-activated receptor gamma (PPARg) [33]. The protein encoded by TOX3 gene may alter chromatin structure by bending and unwinding DNA [34]. In addition, TOX3 can interact with the Cbp/P300 interacting transactivator containing the Glu/Asp rich carboxy-terminal domain 1 (CITED1) and increase its transcription [35]. As a transcription co-regulator, CITED enhances the activity of transcription factors such estrogen receptors[36].The FSHR variant rs2268361 was determined to be related to the ovarian response to FSH [37].Inactivating mutations of FSHR lead to hypergonadotropic hypogonadism and preantral-stage follicle stagnation [37]. HMGA2 is involved in aIGF2 mRNA binding protein 2 (IMP2) pathway, shown to be activated in PCOS patients and capable of promoting the proliferation of granulosa cells [38]. Moreover, HMGA2 is important in modulating glucose transporter type 4 expressions [39]. The YAP1 gene is one of the transcriptional targets of the Hippo pathway, which controls organ size by regulating cell growth, proliferation, and apoptosis [40] while the RAB5B gene is involved in protein trafficking, endocytotic processes and receptor recycling [41-43]. Although many studies have demonstrated the association of specific loci with PCOS in populations of Chinese and European Caucasian ancestry, it is not known whether these loci could contribute to PCOS susceptibility in Saudi women. The aim of this study was to determine in the western Saudi population the presence of common PCOS variants detected previously in Chinese and European Caucasian women. In this population, we also investigated the association between the variants and PCOS clinical symptoms and subgroups.

## Methodology

### Study Design:

The power calculation, inclusion and exclusion criteria for this case-control study have been previously explained [44]. In summary, 95 PCOS patients diagnosed according to the Rotterdam criteria were compared with 94 women with normal ovulation. The clinical and biochemical measures for the diagnosis were described [44]. Patients and controls were recruited either from the Obstetrics and Gynecology Clinics, King Abdulaziz University Hospital or the Centre of Innovation in Personalized Medicine (CIPM), KAU, Jeddah, Saudi Arabia between 2016-2018. The study was approved by the Biomedical Ethics Unit, Faculty of Medicine, KAU (approval number: 407-15), and written informed consent was obtained from participants prior to sample collection. The study was conducted in accordance with the Declaration of Helsinki. The PCOS patients were divided into four subgroups according to their clinical symptoms. Each subgroup was tested individually to investigate whether the associations between SNPs and PCOS were absolute or relative to combined symptoms. After classification of the patients into subgroups, the sample size was 82 as 13 patients were excluded to avoid misleading results as the third symptom (HA) was not investigated (Table 1).

### Genotyping:

The QIAamp DNA Mini Blood Kit (Qiagen, Hilden, Germany) was used to isolate genomic DNA from peripheral whole blood according to the manufacturer's instructions. TaqManTM SNP Genotyping Assays (Thermo Fisher Scientific, Waltham, MA, USA) were used for the genotyping of THADA variant rs13429458 (assay ID: C__30817938_10), TOX3 variant rs4784165 (assay ID: C__30765160_10), FSHR variant rs2268361 (assay ID: C__11813031_1_), YAP1 variant rs1894116 (assay ID:C__11480397_10) , RAB5B variant rs705702 (assay ID:C___3188034_10) , and HMGA2 variant rs2272046 (assay ID: C__15961809_10). Allelic PCR products were analyzed using the Quant Studio 12K Flex Real-Time PCR System (Thermo Fisher Scientific).

### Statistical analysis:

Data analysis was conducted using the IBM SPSS software version 24 (SPSS^™^ Inc., NY, USA). The participants clinical characteristics were expressed as median ± inter quartile range (IQR), and p-values were calculated using the Mann-Whitney test as the data were non-normally distributed. The genotype and allele data were expressed as frequencies. The differences between study groups were analyzed using the chi-squared test. Multinomial logistic regression was used to examine the association of the variants with PCOS clinical variables. Values of p < 0.05 were considered statistically significant.

## Results

All participant clinical parameters are listed in Table 2.

There was a significant link between AMH at the previously determined [45] cut off level (3.19 ng/ml) and FSHR variant rs2268361 in the control group (p = 0.016, Table 5). Multinomial logistic regression showed a significant association between the TT genotype of the rs2268361 variant and normal levels of AMH (<3.19) (OR = 6.2, B = 1.821, p = 0.009). Hence, homozygosity in the T allele ofrs2268361 is potentially protective, associated with normal levels of AMH among non-PCOS women. No relationship was detected between the other clinical parameters including obesity, OA, and PCOM, and the six SNPs using the chi-squared test.

## Allele and genotype frequency:

The genotype distribution and allele frequencies of the six SNPs are listed in Table 3. There was significant relationship between PCOS and THADA rs13429458 (p = 0.033), but no link was detected with the other genetic variants.

## The association of the six variants with PCOS clinical characteristics:

There was a significant relationship between THADA variant rs13429458 and HA phenotype in the PCOS group (p = 0.031, Table 4) by the chi-squared test. Multinomial logistic regression revealed that the AA genotype in THADA variant rs13429458 was positively correlated with higher frequency of HA than the AC genotype (OR = 2.9, B = 1.053, p = 0.026). Therefore, homozygosity in allele A in rs13429458 variant is predicted as a risk genotype associated with HA.

## THADArs13429458 and TOX3rs4784165 variants are correlated to the OA+PCOM subgroup:

The PCOS group was divided into four subgroups according to the clinical symptoms. There was significant correlation between the THADA rs13429458 and TOX3 rs4784165 SNPs and the OA+PCOM subgroup (p = 0.009, p = 0.028 respectively, Table 6).

## HMGA2 rs2272046 variant is correlated to HA+OA subgroup:

The HMGA2 variant rs2272046 showed a significant correlation with the HA+OA subgroup (p = 0.043, Table 6). No other correlations were detected among other variants within PCOS subgroups.

## Discussion

In the last two decades, numerous studies have focused on the genetic pathogenesis of PCOS in order to understand the genetic predisposition to the disorder. In the present study, we examined six previously reported PCOS-associated SNPs identified collectively through GWAS in populations of Chinese Han and European ancestry [23, 26,28-32] to investigate their association in the Saudi population.

Of the six SNPs, only the THADA rs13429458 variant was associated with PCOS. This was also found in previous studies of populations with Chinese and European ancestry [23,26,28]. The same association was found in a study of the Hainan Chinese population [46]. Furthermore, a family-based analysis of PCOS susceptibility loci on chromosome 2p21 in the Han Chinese population showed a significant association of rs13429458 with risk of PCOS [47]. Two studies of Caucasian patients concluded that the genotype distribution of rs13429458 did not differ significantly between the patient and control groups [48,49]. In contrast, two studies of European populations showed no association between rs13429458 and PCOS [30,32].

The thyroid adenoma-associated protein encoded by the THADA gene is expressed in many organs [33].One GWAS reported the association of THADA with type 2 diabetes particularly through a probability effect on pancreatic beta-cell function [50]. As a result, such a protein would be expected to affect various body processes, not unlike PCOS, which is characterized by dysfunction in multiple organ systems. In the present study, it was correlated in PCOS women with the HA phenotype. This may provide clues to the role of rs13429458 in the etiology of PCOS, as HA is one of the clinical symptoms of PCOS. Previously, the AA genotype for rs13429458 in THADA was detected in different phenotypes to be associated with increased LH, testosterone levels, and the LH/FSH ratio in subjects with PCOS [51]. Moreover, the genotype frequency distribution of rs13429458 was not influenced by hirsutism or increased metabolic parameters, including fasting glucose and insulin level [48].

It was demonstrated that the AMH receptors expressed in the brain may be involved in initiation of gonadotropin-releasing hormone (GnRH) release from hypothalamic neurons [52]. GnRH causes the pituitary gland in the brain to make and secrete FSH [53] which may explain our finding correlation of the FSHR rs2268361 variant and AMH at cutoff level in the control group. Furthermore, FSH, as well as AMH, contributes to follicle development, and AMH preserves the follicles in the primordial stage, estimates the number of ovum in the ovaries, thereby indicating the ovary reserve [54,55]. In PCOS,FSH-the principal regulator of follicular growth and maturation [56]-is suppressed below the level needed in the early follicular phase to stimulate normal follicle maturation. As a result, the development of large antral follicles (5-8 mm) is arrested [57]. The arrested antral follicles will increase serum AMH in PCOS women due to greater production of AMH per follicle [58,59].

The PCOS subgroups differed significantly on almost all anthropometric, endocrine, and metabolic characteristics [60]. Thus, we analyzed the association of each subgroup separately with the SNPs to determine the variations with different clinical variables. Accordingly, we detected significant correlation of THADA rs13429458 variant, TOX3 rs4784165 variant with the OA+PCOM subgroup, and HMGA2 rs2272046 variant with the HA+PCOM subgroup.TOX3 and HMGA2 genes are well-known contributors to PCOS through enhancement of the activity of transcription factors such as the estrogen receptor [36] and promoting the proliferation of granulosa cells [38], respectively. No significant association of PCOS or its phenotypes with FSHR rs2268361, YAP1rs1894116, or RAB5B rs705702 in the Saudi population, which could be attributed to the small sample size.

## Conclusion

We report the link between the THADA rs13429458 gene variant and PCOS in western population. We also document the link of THADA rs13429458 and TOX3 rs4784165 variants with combined OA and PCOM phenotype of PCOS patients. It is further noted that the HMGA2 rs2272046 variant is linked with combined HA and PCOM phenotypes. These observations should be further verified using large GWAS to delineate the polygenic risk in PCOS among Saudi population. 

## Funding:

This study was funded by the Center of Innovation in Personalized Medicine (CIPM), King Abdulaziz University, Jeddah, Saudi Arabia.

## Author's contribution:

S.B. conceptualized the study. S.B. and N.A. performed experiments, analyzed data, and wrote the manuscript. S.B. corrected the final version of the manuscript. All authors read and approved the final manuscript.

## Figures and Tables

**Table 1:** Classification of PCOS into groups according to clinical symptoms

PCOS Subgroup	Frequency
Full PCOS (HA + OA + PCOM)	42
Non-PCOM (HA + OA)	6
Non-hyperandrogenic (OA + PCOM)	25
Ovulatory (HA +PCOM)	9
Total samples	82
HA: hyperandrogenism. OA: oligo/amenorrhea. PCOM: polycystic ovarian morphology.

**Table 2:** Clinical characteristics of PCOS patients and control subjects

Variable	Control (n=94)	PCOS patients (n=95)	p-value
Age (years)	21.0± 3	22.0± 9.0	0.015*
BMI (kg/m^2^	22.7± 5.9	24.56± 7.34	0.003**
LH (IU/ml)	5.7± 5.7	9.0± 8.7	0.001**
FSH (IU/ml)	4.6± 2.6	4.8± 2.4	0.339
LH/FSH ratio	1.2± 1.5	1.9± 1.6	0.001**
AMH (ng/ml)	2.3± 1.4	4.8± 4.76	<0.0001***
The values are expressed as median ± IQR, p-values were calculated using the Mann-Whitney test for non-normal distribution data. p-value <0.05 is statistically significant. BMI: body mass index; LH: luteinizing hormone; FSH: follicle stimulating hormone; AMH: anti-Mullerian hormone. *p<0.05, **p<0.01, ***p<0.001

**Table 3:** Genotype and allele distributions of the six SNPs

	THADA rs13429458				p-value
Genotype frequency	AA	AC		CC	
PCOS (n=95)	61 (64.2%)	33 (34.7%)		1 (1.1%)	0.033*
Control (n=94)	74 (78.7%)	18 (19.2%)		2 (2.1%)	
Total =189	135 (71.4%)	51 (27%)		3 (1.6%)	
Allele frequency	A		C		
PCOS (n=95)	155 (81.6%)		35 (18.4%)		
Control (n=94)	166 (88.3%)		22 (11.7%)		
	TOX3rs4784165				p-value
Genotype frequency	GG	GT		TT	
PCOS (n=94)	12 (12.8%)	35 (37.2%)		47 (50%)	0.606
Control (n=94)	8 (8.5%)	41 (43.6%)		45 (47.9%)	
Total =188	20 (10.6%)	76 (40.4%)		92 (48.9%)	
Allele frequency	G		T		
PCOS (n=95)	59 (31.4%)		129 (68.6 %)		
Control (n=94)	57 (30.3%)		131 (69.7%)		
	FSHRrs2268361				p-value
Genotype frequency	CC	CT		TT	
PCOS (n=95)	22 (23.2%)	47 (49.5%)		26 (27.3%)	0.339
Control (n=94)	20 (21.3%)	39 (41.5%)		35 (37.2%)	
Total =189	42 (22.2%)	86 (45.5%)		61 (32.3%)	
Allele frequency	C		T		
PCOS (n=95)	91 (47.9%)		99 (52.1%)		
Control (n=94)	79 (42%)		109 (58%)		
	YAP1rs1894116				p-value
Genotype frequency	AA	AG		GG	
PCOS (n=95)	83 (87.3%)	9 (9.5%)		3 (3.2%)	0.266
Control (n=94)	78 (82.9%)	15 (16%)		1 (1.1%)	
Total =189	161 (85.2%)	24 (12.7%)		4 (2.1%)	
Allele frequency	A		G		
PCOS (n=95)	175 (92.1%)		15 (7.9%)		
Control (n=94)	171 (91%)		17 (9%)		
	RAB5Brs705702				p-value
Genotype frequency	AA	AG		GG	
PCOS (n=95)	70 (73.6%)	22 (23.2%)		3 (3.2%)	0.641
Control (n=94)	65 (69.2%)	27 (28.7%)		2 (2.1%)	
Total =189	135 (71.4%)	49 (25.9%)		5 (2.6%)	
Allele frequency	A		G		
PCOS (n=95)	162 (85.3%)		28 (14.7%)		
Control (n=94)	157 (83.5%)		31 (16.5%)		
SNP	HMGA2rs2272046				p-value
Genotype frequency	AA	AC		CC	
PCOS (n=95)	91 (95.8)	4 (4.2%)		0 (0%)	0.414
Control (n=94)	92 (97.9%)	2 (2.1%)		0 (0%)	
Total =189	183 (96.8%)	6 (3.2%)		0 (0%)	
Allele frequency	A		C		
PCOS (n=95)	186 (97.9%)		4 (2.1%)		
Control (n=94)	186 (98.9%)		2 (1.1%)		
p-values were calculated by Pearson's chi-squared test. *p-values < 0.05

**Table 4:** Relationship between the six variants and HA phenotype in the PCOS group

SNPs	Gene	Genotype	Frequency With HA/ without HA	p-value
rs13429458	*THADA*	AA	43/15	0.031*
		AC	15/15	
		CC	0/1	
rs4784165	*TOX3*	GG	6-Jun	0.344
		GT	24/9	
		TT	27/16	
rs2268361	*FSHR*	CC	9-Dec	0.401
		CT	33/13	
		TT	13/9	
rs1894116	*YAP1*	AA	51/26	0.816
		AG	4-May	
		GG	1-Feb	
rs705702	*RAB5B*	AA	42/24	0.871
		AG	14/6	
		GG	1-Feb	
rs2272046	*HMGA2*	AA	55/30	0.673
		AC	1-Mar	
		CC	0/0	
The p-values were calculated by chi-squared test. HA: hyper androgenism. *p-values < 0.05

**Table 5:** Relationship between the six variants and AMH cutoff level in PCOS and control groups

SNPs	Gene	Tested group	Genotype	Frequency	p-value
				AMH>3.19/	
				AMH<3.19	
rs13429458	*THADA*	PCOS	AA	34/14	0.799
		(n=79)	AC	22/8	
			CC	Jan-00	
		Control	AA	18/36	0.321
		(n=69)	AC	12-Mar	
			CC	0/0	
rs4784165	*TOX3*	PCOS	GG	4-Jun	0.52
		(n=78)	GT	20/9	
			TT	30/9	
		Control	GG	4-Jan	0.498
		(n=69)	GT	18-Nov	
			TT	26-Sep	
rs2268361	*FSHR*	PCOS	CC	13/5	0.293
		(n=79)	CT	27/14	
			TT	17/3	
		Control	CC	11-Apr	0.016*
		(n=69)	CT	13/13	
			TT	24-Apr	
rs1894116	*YAP1*	PCOS	AA	49/19	0.466
		(n=79)	AG	3-May	
			GG	Mar-00	
		Control	AA	17/42	0.51
		(n=69)	AG	5-Apr	
			GG	0/1	
rs705702	*RAB5B*	PCOS	AA	43/16	0.298
		(n=79)	AG	13/4	
			GG	2-Jan	
		Control	AA	15/32	0.708
		(n=69)	AG	15-May	
			GG	1-Jan	
rs2272046	*HMGA2*	PCOS	AA	54/22	0.273
		(n=79)	AC	Mar-00	
			CC	0/0	
		Control	AA	21/47	0.505
		(n=69)	AC	0/1	
			CC	0/0	
The p-values were calculated by chi-squared test. *p-values <0.05

**Table 6:** The correlation of THADA rs13429458,TOX3 rs4784165, and HMGA2 rs2272046 with PCOS subgroups

SNPs	Gene	Subgroup	Genotype	PCOS subgroup frequency	p-value
rs13429458	*THADA*	OA+PCOM	A/A	12 (48%)	0.009**
		(n=25)	A/C	12 (48%)	
			C/C	1 (2%)	
rs4784165	*TOX3*	OA+PCOM	G/G	6 (24%)	0.028*
		(n=25)	G/T	5 (20%)	
			T/T	14 (56%)	
rs2272046	*HMGA2*	HA+OA	A/A	5 (83.3%)	0.043*
		(n=6)	A/C	1 (16.7%)	
			C/C	0 (0%)	
p-values were calculated by chi-squared test. *p-values < 0.05, **p-values < 0.01. HA: hyper androgenism; OA: Oligo/amenorrhea; PCOM: polycystic ovarian morphology.
